# Phospholipase C and myosin light chain kinase inhibition define a common step in actin regulation during cytokinesis

**DOI:** 10.1186/1471-2121-8-15

**Published:** 2007-05-17

**Authors:** Raymond Wong, Lacramioara Fabian, Arthur Forer, Julie A Brill

**Affiliations:** 1 Program in Developmental and Stem Cell Biology, The Hospital for Sick Children, TMDT Building, East Tower, 101 College St., Rm. 13-307, Toronto, Ontario M5G 1L7, Canada; 2 Institute of Medical Science, University of Toronto, Toronto, Ontario, M5S 1A8, Canada; 3 Department of Biology, York University, Toronto, Ontario M3J 1P3, Canada

## Abstract

**Background:**

Phosphatidylinositol 4,5-bisphosphate (PIP_2_) is required for successful completion of cytokinesis. In addition, both PIP_2 _and phosphoinositide-specific phospholipase C (PLC) have been localized to the cleavage furrow of dividing mammalian cells. PLC hydrolyzes PIP_2 _to yield diacylglycerol (DAG) and inositol trisphosphate (IP_3_), which in turn induces calcium (Ca^2+) ^release from the ER. Several studies suggest PIP_2 _must be hydrolyzed continuously for continued cleavage furrow ingression. The majority of these studies employ the N-substituted maleimide U73122 as an inhibitor of PLC. However, the specificity of U73122 is unclear, as its active group closely resembles the non-specific alkylating agent N-ethylmaleimide (NEM). In addition, the pathway by which PIP_2 _regulates cytokinesis remains to be elucidated.

**Results:**

Here we compared the effects of U73122 and the structurally unrelated PLC inhibitor ET-18-OCH_3 _(edelfosine) on cytokinesis in crane-fly and *Drosophila *spermatocytes. Our data show that the effects of U73122 are indeed via PLC because U73122 and ET-18-OCH_3 _produced similar effects on cell morphology and actin cytoskeleton organization that were distinct from those caused by NEM. Furthermore, treatment with the myosin light chain kinase (MLCK) inhibitor ML-7 caused cleavage furrow regression and loss of both F-actin and phosphorylated myosin regulatory light chain from the contractile ring in a manner similar to treatment with U73122 and ET-18-OCH_3_.

**Conclusion:**

We have used multiple inhibitors to examine the roles of PLC and MLCK, a predicted downstream target of PLC regulation, in cytokinesis. Our results are consistent with a model in which PIP_2 _hydrolysis acts via Ca^2+ ^to activate myosin via MLCK and thereby control actin dynamics during constriction of the contractile ring.

## Background

Cell proliferation and growth require the coordination of cell signaling pathways. PLC plays an important role in cell signaling, mediating transduction of signals from a variety of intracellular and extracellular stimuli [1,2]. PLC-dependent hydrolysis of PIP_2 _produces the second messengers DAG and IP_3 _(reviewed in [3]). IP_3 _binds specific receptors on the ER to mobilize calcium (Ca^2+) ^from internal stores. DAG and Ca^2+ ^activate protein kinase C (PKC), which stimulates cell growth [4]. In addition, Ca^2+ ^itself facilitates diverse cellular events including membrane trafficking, contractility and proliferation [5]. PLC-dependent pathways thus play key roles in promoting cell growth.

**Figure 1:**
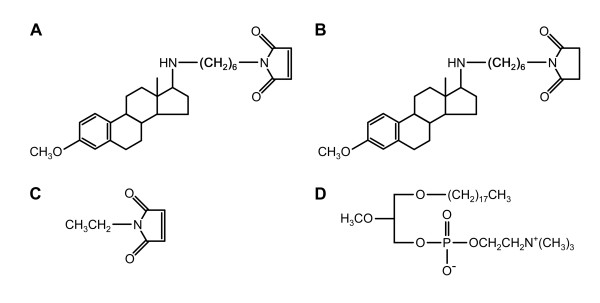
**Chemical structures of PLC inhibitors and controls**. Chemical structures of (A) U73122, (B) U73343, (C) NEM and (D) ET-18-OCH_3_.

The synthetic aminosteroid U73122 (Figure 1A) is an important tool in identifying and studying PLC-dependent processes. Initially discovered in a search for inhibitors of platelet activation, U73122 was found to inhibit PLC function [6]. U73122 causes decreases in IP_3 _and DAG production, calcium levels, and phosphatidylinositol (PI) turnover in agonist-stimulated platelets, indicating that U73122 blocks PLC-mediated hydrolysis of PIP_2 _in treated cells [7]. The mechanism of action of U73122 is currently unknown [8], although an examination of its structure can provide some insight into the biologically active domains of the molecule. The inhibitory activity of U73122 can be reduced by alteration of the C17 side chain or removal of the 3-methoxy group [7]. Substitution of the electrophilic maleimide group of U73122 with the less electrophilic succinimide produces the inactive isomer U73343, which differs from the active form only by the absence of a double bond on the pyrrole ring (Figure 1B). Thus, reactivity appears to reside largely in the NEM moiety of the molecule. NEM (Figure 1C) is a sulfhydryl alkylating agent that covalently modifies cysteine residues, raising the possibility that U73122 acts by a similar mechanism.

Using inhibitors affecting PI metabolism, we previously showed that continuous PI cycling is required for cytokinesis [9]. In particular, treatment of cells with U73122 resulted in regression of the cleavage furrow and failure of cytokinesis [9,10]. The effects of U73122 suggested PIP_2 _hydrolysis is required for normal cytokinesis. Indeed, cytokinesis failed in cells treated with an IP_3 _receptor antagonist or in which intracellular Ca^2+ ^was chelated by BAPTA-AM [10]. Perhaps most strikingly, our data indicated that PIP_2 _hydrolysis may be required to maintain F-actin in the contractile ring [9].

U73122 may exert non-specific effects, however, because several lines of evidence suggest that U73122 may affect phosphoinositide cycling or Ca^2+ ^levels independent of PLC inhibition. In rabbit platelets, U73122 caused up to 50% reduction in the levels of phosphatidylinositol 4-phosphate (PIP) and PIP_2_, but not PI, indicating that U73122 may inhibit both PI and PIP kinases. Although IP_3 _levels were reduced in agonist-stimulated cells, consistent with inhibition of PLC, this may have been an indirect effect of a shortage in substrate availability [11]. In U73122-treated mouse fibroblasts and rat neutrophils, inhibition of Ca^2+ ^influx across the plasma membrane contributed to the suppression of Ca^2+ ^levels [12,13]. In contrast, U73122 actually increased Ca^2+^ release from internal stores in rat pancreatic acinar cells and rat liver microsomes [14,15].

A second PLC inhibitor, the lysophosphatidylcholine analogue ET-18-OCH_3 _(Figure 1D; [16]), is of interest in cancer therapy because of its demonstrated anti-tumor properties [17]. ET-18-OCH_3 _and related compounds inhibit phosphatidylinositol 3-kinases and PKC [18-21] and selectively promote tumor cell apoptosis [17,22]. Like U73122, ET-18-OCH_3 _also has other off-target effects, including release of Ca^2+ ^from intracellular stores [23]. However, unlike U73122, ET-18-OCH_3 _is inserted in the plasma membrane, and its effects may be due in part to alteration of membrane lipid and protein composition [24-26]. In both human histiocytic lymphoma and tumorigenic rat liver cells, ET-18-OCH_3 _inhibits cytokinesis [27,28].

To determine if U73122 inhibits cytokinesis via PLC, we compared the effects of U73122 with those of ET-18-OCH_3 _and NEM. We further examined the role of a potential downstream target of PLC activity, MLCK. We found that inhibition of MLCK perturbed cytokinesis in a manner similar to inhibition of PLC by U73122 and ET-18-OCH_3_. Our results are consistent with a model in which PLC and MLCK act in the same pathway to maintain the integrity of the contractile ring, implicating MLCK as a target of Ca^2+ ^regulation in this process.

## Results

### PLC inhibition causes cleavage furrows to regress

**Figure 2:**
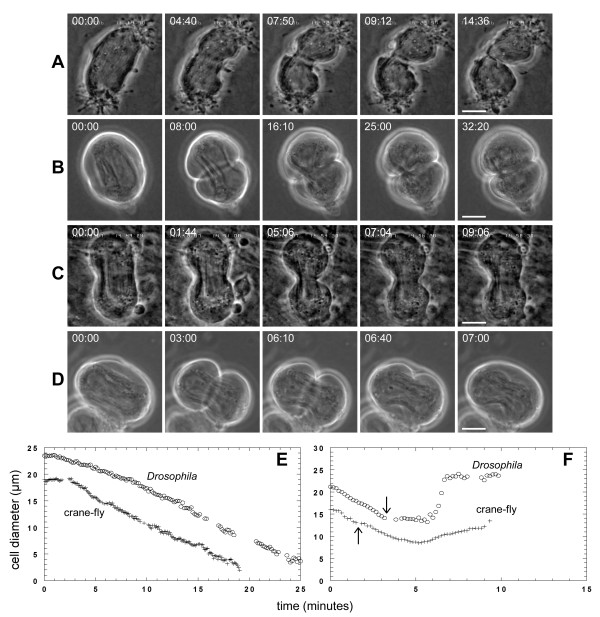
**U73122 causes cleavage furrow regression in crane-fly and *Drosophila *spermatocytes**. (A, B) Phase-contrast images showing time-course of cytokinesis in untreated (A) crane-fly and (B) *Drosophila *spermatocytes [see Additional files 1, 2]. Times are in min:sec. Bars, 10 μm. (C, D) Phase-contrast time-course of a (C) crane-fly and (D) *Drosophila *spermatocyte treated with U73122 during cytokinesis [see Additional files 3, 4]. U73122 was added immediately after the time-point depicted in the second panel. Crane-fly and *Drosophila *spermatocytes were treated with 4.3 μM and 10 μM U73122 respectively. Note that the crane-fly spermatocyte was followed for only a short time after treatment (for a longer time-course, see [9]). Bars, 10 μm. (E) Plot of the change in cell diameter (ordinate) over time (abscissa) for the treated crane-fly (+) and *Drosophila *(o) spermatocytes shown in A, B. (F) Plot of the change in cell diameter (ordinate) over time (abscissa) for the crane-fly (+) and *Drosophila *(o) spermatocytes shown in C, D. Arrows indicate time of drug treatment.

We previously showed that the PLC inhibitor U73122 caused cleavage furrow regression during cytokinesis in crane-fly and *Drosophila melanogaster *spermatocytes [9,10]. In control cells, cytokinesis was initiated by invagination of the equatorial plasma membrane to form a furrow between the nascent daughter cells (Figure 2A, B) [see Additional files 1, 2] [10,29]. Ingression continued unabated until the cleavage furrow reached a minimum diameter of one to several micrometers (Figure 2E). When added after initiation of furrowing, U73122 arrested cytokinesis and caused cleavage furrow regression (Figure 2C, D, F) [see Additional files 3, 4] [9,10]. During cleavage, mitochondria and parafusorial membranes along the spindle become constricted as the plasma membrane ingressed at the equator [10]. In *Drosophila *spermatocytes treated with U73122, these structures remained constricted as the plasma membrane regressed, and often underwent continued constriction of several micrometres after regression of the furrow (Figure 2D). The effects of U73122 were not reversible at any concentration in *Drosophila*; however, in crane-fly spermatocytes, the effects at the minimum effective dosage of U73122 were reversed after washing [9].

**Figure 3:**
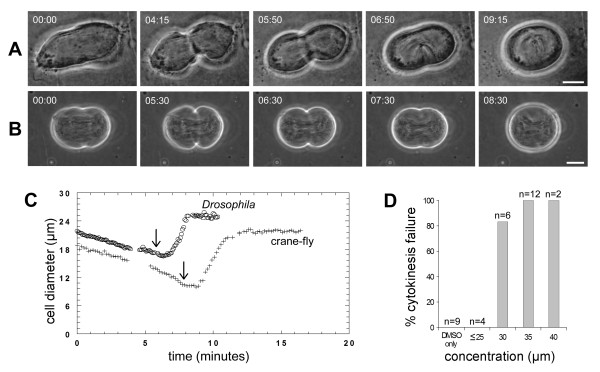
**ET-18-OCH_3 _causes cleavage furrow regression in crane-fly and *Drosophila *spermatocytes**. (A, B) Time-lapse phase-contrast micrographs showing ET-18-OCH_3_-treated dividing (A) crane-fly or (B) *Drosophila *spermatocytes [see Additional files 5, 6]. For each cell, 35 μM ET-18-OCH_3 _was added just after the time-point depicted in the second panel. Bars, 10 μm. (C) Plot of the change in cell diameter (ordinate) over time (abscissa) for the crane-fly (+) and *Drosophila *(o) spermatocytes shown in A, B. Arrows indicate time of drug addition. (D). Sensitivity of *Drosophila *spermatocyte cytokinesis to increasing concentrations of ET-18-OCH_3_. Cytokinesis failed in the majority of cells treated with 30 μM and in all cells treated with 35–40 μM ET-18-OCH_3_.

To determine if a second PLC inhibitor had a similar effect, we treated dividing crane-fly and *Drosophila *spermatocytes with ET-18-OCH_3_. In both species, ET-18-OCH_3 _inhibited cytokinesis and caused the cleavage furrow to regress (Figure 3A, B, C) [see Additional files 5, 6]. These effects were seen at concentrations of 30 μM or greater in *Drosophila *spermatocytes (Figure 3D) and 35 μM or greater in crane-fly spermatocytes (data not shown). In crane-fly spermatocytes, but not in *Drosophila *spermatocytes, cleavage furrow regression was reversible in 2/4 treated cells. Thus U73122 and ET-18-OCH_3 _have similar effects on cytokinesis.

### Alkylation stops furrow ingression but does not cause regression

**Figure 4:**
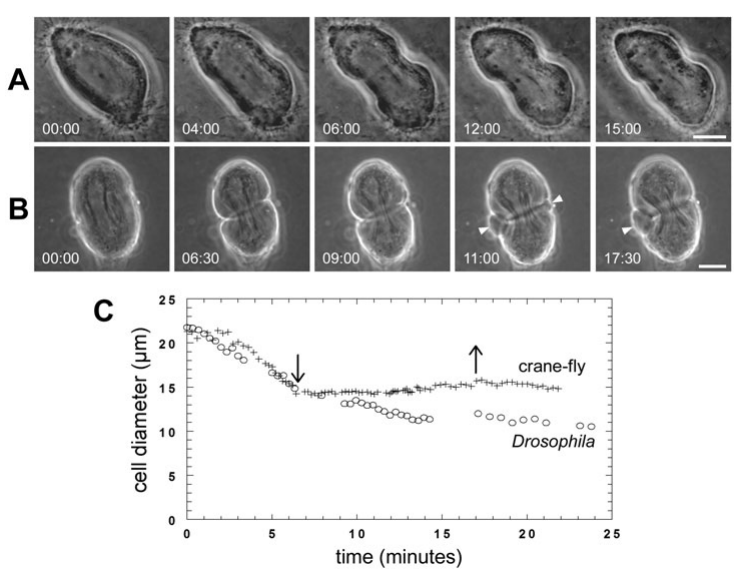
**Treatment with NEM blocks cytokinesis but does not cause cleavage furrow regression**. (A, B) Time-lapse phase-contrast images showing a dividing (A) crane-fly spermatocyte treated with 50 μM NEM [see Additional file 7] or (B) *Drosophila *spermatocyte treated with 10 μM NEM. In both experiments, cells were treated just after the time-point depicted in the second panel. Note the appearance of blebs after treatment of the *Drosophila *spermatocyte (arrows) [see Additional file 8]. Bars, 10 μm. (C) Plot of the change in cell diameter (ordinate) over time (abscissa) for the crane-fly (+) and *Drosophila *(o) spermatocytes shown in A, B. NEM was added at the time-point indicated by the left arrow. In the crane-fly spermatocyte, cleavage remained arrested even after washing with Insect Ringers (right arrow).

To determine if the effects of U73122 were due to non-specific alkylation of off-target proteins, we directly examined the effects of the alkylating agent NEM on cytokinesis. In both crane-fly and *Drosophila *spermatocytes, NEM stopped cytokinesis but did not cause regression of the cleavage furrow (Figure 4A, B) [see Additional files 7, 8]. The effective concentration differed between the two species. In crane-fly spermatocytes, NEM inhibited furrowing at concentrations of 50 μM or greater. Cleavage arrest occurred immediately after treatment and cleavage furrows did not regress or resume constriction even after the drug was washed out (Figure 4C). In *Drosophila *spermatocytes, NEM also irreversibly stopped furrowing, although it was effective at concentrations as low as 500 nM (Figure 4B, C). In addition, NEM caused large blebs to form on the plasma membrane (Figure 4B, arrowheads) [see Additional file 8]. NEM did not cause regression of cleavage furrows at any concentration tested (up to 100 μM).

### Myosin Light Chain Kinase (MLCK) inactivation causes cleavage furrows to regress

**Figure 5:**
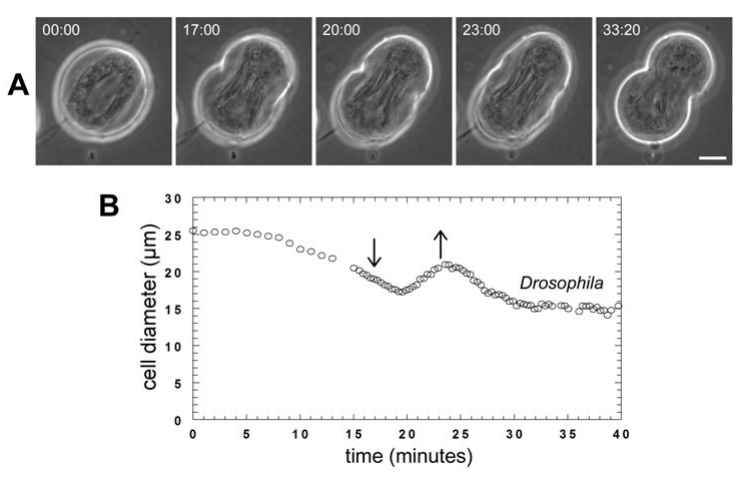
**Treatment with ML-7 causes cleavage furrow regression**. (A) Time-course of a dividing *Drosophila *spermatocyte treated with 80 μM ML-7 just after the time-point depicted in the second panel. The cell was washed with Insect Ringer's buffer just after the time-point depicted in the fourth panel. Bar, 10 μm. (B) Plot of the change in cell diameter (ordinate) over time (abscissa) for the *Drosophila *spermatocyte shown in A. Time-points of ML-7 addition (left arrow) and washout (right arrow) are indicated [see Additional file 9].

To test if IP_3 _receptor-mediated Ca^2+ ^release activates MLCK during cytokinesis [10], we examined the effects of ML-7, an inhibitor of MLCK and hence of myosin activation. A concentration range of 75–80 μM was chosen because lower concentrations had little or no effect on cytokinesis ([29]; not shown). In both crane-fly and *Drosophila *spermatocytes, treatment with 75 μM (crane-fly) or 80 μM (*Drosophila*) ML-7 caused cleavage furrow regression ([29]; Figure 5A) [see Additional file 9]. Treatment of *Drosophila *spermatocytes with ML-7 was reversible upon washout (Figure 5B) [see Additional file 9], unlike treatment with U73122, ET-18-OCH_3 _or NEM; however, after resuming, ingression arrested prematurely in 21 out of 33 cells. Thus, the effects of ML-7 on cytokinesis in *Drosophila *spermatocytes are identical to those previously reported in crane-fly spermatocytes [29]. That ML-7 treatment blocks cytokinesis in a manner similar to U73122 and ET-18-OCH_3 _is consistent with PLC and MLCK acting in the same pathway.

### PLC and MLCK activities are required to maintain actin at the cleavage furrow

**Figure 6:**
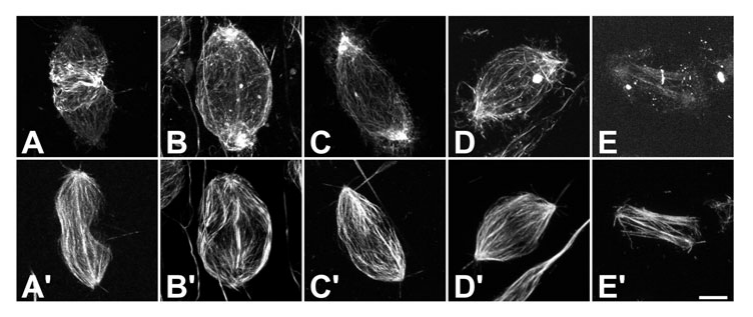
**F-actin relocalizes from the contractile ring to the poles upon inhibition of PLC or MLCK**. Confocal images of dividing crane-fly spermatocytes stained for (A-E) F-actin and (A'-E') tyrosinated tubulin. (A, A') Untreated control cell. In untreated cells, F-actin was concentrated in the contractile ring (A) and tubulin in the spindle (A'). (B, B') Cell treated with U73122. (C, C') Cell treated with ET-18-OCH_3_. (D, D') Cell treated with ML-7. Cells treated with these inhibitors had reduced contractile ring F-actin and accumulation of F-actin at the poles (B-D). (E, E') Cell treated with NEM. Note small band of F-actin at the equator, as well as reduced F-actin density throughout the rest of the cell. Also note loss of astral microtubules (E'). Bar, 10 μm.

To investigate the effect of the various inhibitors on cytoskeletal components, we stained crane-fly spermatocytes for F-actin and tubulin. In control cells, actin filaments were arranged in a circumferential band around the equator of the cell, with very little F-actin at the poles. Tubulin was present in spindle and astral microtubules and was organized longitudinally from pole-to-pole (Figure 6A, A'). After treatment with U73122, tubulin organization was similar to that of control cells. However, actin filaments became much shorter and more disorganized, with many cells showing reduction in F-actin at the cleavage furrow concomitant with accumulation of F-actin at the poles (Figure 6B, B'; see also [9]). Similarly, after ET-18-OCH_3 _treatment, F-actin accumulated at the poles, with the few actin filaments at the equator appearing more sparsely packed than in control cells (Figure 6C, C'). After treatment with ML-7, the distribution of F-actin and tubulin closely resembled treatment with U73122 and ET-18-OCH_3_: tubulin localization was unchanged as compared to control cells, whereas F-actin disappeared from the contractile ring and accumulated at the poles. These actin filaments were longer than in U73122-treated cells and appeared more disorganized (Figure 6D, D'). In contrast, treatment with NEM had an entirely different effect: astral microtubules were absent and F-actin in the contractile ring was dramatically reduced, with the majority of cells (5/7) showing a compact band of F-actin at the equator. In addition, F-actin did not accumulate at the poles. Actin filaments in the cortex were broken into small fragments or completely depolymerized (Figure 6E, E').

### PLC and MLCK activities are required to maintain activated myosin

**Figure 7:**
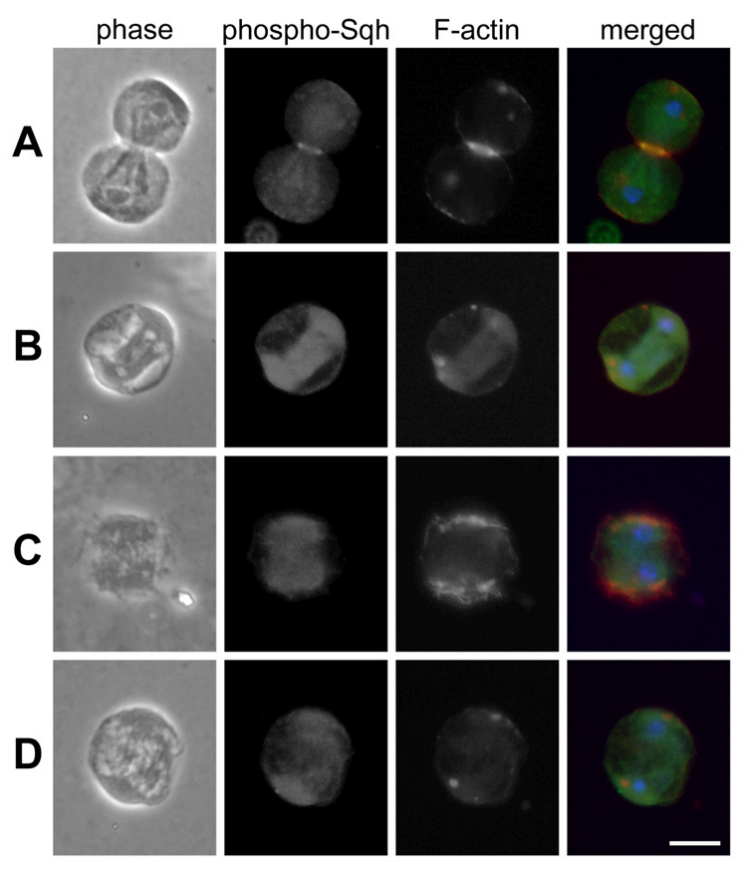
**Inhibition of PLC or MLCK causes loss of phosphorylated myosin regulatory light chain from the contractile ring**. Phase-contrast and immunofluorescence images of dividing *Drosophila *spermatocytes stained for phospho-Sqh (green), F-actin (red) and DNA (blue). (A) Untreated control cell. Note colocalization of phospho-Sqh and F-actin in the contractile ring. (B) Cell treated with U73122. (C) Cell treated with ET-18-OCH_3_. (D) Cell treated with ML-7. (B-C) In cells treated with these inhibitors, phospho-Sqh and F-actin were no longer evident at the equator, whereas F-actin was relocalized to the poles. Bar, 10 μm.

To directly test if these inhibitors interfered with phosphorylation of myosin regulatory light chain (Sqh in *Drosophila*; [30]), a known target of MLCK, we stained dividing *Drosophila *spermatocytes for phosphorylated Sqh (phospho-Sqh; [31]) and F-actin. In control cells, phospho-Sqh strongly colocalized with F-actin in the contractile ring, weakly colocalized with F-actin on the cell cortex at the poles, and was found weakly colocalized with the mitochondria and parafusorial membranes (Figure 7A). In contrast, cells treated with U73122, ET-18-OCH_3 _or ML-7 no longer showed evidence of phospho-Sqh or F-actin in the contractile ring. Instead, phospho-Sqh remained associated with the mitochondria and parafusorial membranes, whereas F-actin accumulated at the poles of the cell (Figure 7B–D).

## Discussion

U73122 is widely accepted as a specific inhibitor of PLC. Nevertheless, its mechanism of action has yet to be elucidated. In some systems, NEM mimics treatment with U73122 [32], raising the possibility that previously reported effects of U73122 were due to alkylation of off-target proteins rather than inhibition of PLC. To obtain such effects with NEM, Horowitz *et al*. treated cells with concentrations of NEM forty-times larger than those used for U73122. The authors suggested that higher concentrations of NEM were required because NEM lacks the lipophilic steroid domain of U73122 that targets the NEM moiety to membranes [32]. Consistent with their observations, we found that the concentration required for NEM to block cytokinesis in crane-fly spermatocytes was twenty-times greater than the minimum effective concentration of U73122. However, unlike U73122, NEM did not cause regression of the cleavage furrow or redistribution of F-actin to the poles of the cell. In addition, the effect of U73122 on cytokinesis was reversible in crane-fly spermatocytes at the minimum effective concentration [9], whereas the effect of NEM was not. In *Drosophila *spermatocytes, on the other hand, NEM stopped cleavage furrow ingression at a concentration well below the effective concentration of U73122 [10], suggesting that NEM has more pleiotropic effects. Indeed, NEM treatment resulted in blebbing of the plasma membrane, an effect not seen after treatment with U73122. These observations show that the effects of U73122 are distinct from those of NEM, and suggest that they are due to inhibition of PLC by U73122, although our data do not rule out a mechanism in which U73122 inhibits PLC by alkylation. Effects of other inhibitors strengthen the argument that a PLC-dependent pathway is involved in cytokinesis.

A second PLC inhibitor, ET-18-OCH_3_, had effects on cytokinesis similar to U73122. ET-18-OCH_3 _is believed to act by inserting into cellular membranes. Like U73122, ET-18-OCH_3 _caused cleavage furrow regression and redistribution of F-actin to the poles of the cell. These inhibitors have two known overlapping cellular effects: inhibition of PLC and release of Ca^2+ ^from intracellular stores. In previous experiments using *Drosophila *spermatocytes, we showed that treatments predicted to greatly influence intracellular Ca^2+ ^levels had little effect on cytokinesis. Treatment of cells with Ca^2+ ^ionophores A23187 or ionomycin did not block cleavage, although they did induce cell contractility and occasional ectopic cell fusion events [10]. In contrast, chelation of intracellular Ca^2+ ^with the cell permeable chelator BAPTA-AM did block cytokinesis, but only when cells were cultured in buffer lacking Ca^2+ ^[10]. Thus, at least *Drosophila *spermatocytes can divide provided they have either an intracellular or an extracellular source of Ca^2+^, rendering it unlikely that any effect of ET-18-OCH_3 _or U73122 on intracellular Ca^2+ ^stores would have blocked cytokinesis in these cells. The simplest explanation for the common effect of U73122 and ET-18-O-CH_3 _is that PLC activity is required for cytokinesis.

The requirement for PLC activity in cytokinesis appears to be conserved. Several mammalian PLC isoforms, PLCδ1, PLCβ1 and PLCδ3, were found to localize to the cleavage furrows of HeLa, NIH3T3 and MDCK cells during cytokinesis [33,34]. Moreover, PLCγ is tyrosine phosphorylated, and presumably activated, in dividing sea urchin embryos [35]. U73122, which blocks cleavage in sea urchin embryos [35], and ET-18-OCH_3_, which was originally found to interfere with cytokinesis in transformed cells and tumor cell lines [27,28], were recently found to inhibit cytokinesis in NIH3T3 cells [34]. Although there are currently no genetic studies showing a role for individual PLCs in cytokinesis in any system, this is likely due to redundancy, as most organisms contain multiple PLCs.

In dividing crane-fly and *Drosophila *spermatocytes, two PLC inhibitors, U73122 and ET-18-OCH_3_, cause cleavage furrow regression ([9][10]; this study). Inhibition of PLC would be expected to result in an increase in PIP_2_, concomitant with a decrease in the second messengers DAG and IP_3_. PIP_2 _is required for cytokinesis in *Drosophila *spermatocytes and mammalian cells [10,36,37]. However, it is unclear if an increase in PIP_2 _would be deleterious to the cell. On the other hand, inhibition of the IP_3 _receptor with 2-APB or chelation of Ca^2+ ^with BAPTA-AM blocked continued cleavage in *Drosophila *spermatocytes and zebrafish embryos [10,38,39]. Furthermore, we previously showed that treatment of spermatocytes with a Ca^2+ ^ionophore prevented the cells from responding to U73122, strongly suggesting Ca^2+ ^is a key downstream effector of PIP_2 _hydrolysis during cytokinesis [10]. Effects of the MLCK inhibitor ML-7 confirm this interpretation.

MLCK, a potential target of Ca^2+ ^during cytokinesis, phosphorylates myosin regulatory light chain on Ser-19, and to a lesser extent on Thr-18. This diphosphorylation activates non-muscle myosin II, allowing it to form bipolar thick filaments postulated to constrict F-actin in the contractile ring [40-42]. Although recent experiments have focused on roles for other myosin activating kinases, Rho kinase and citron kinase, in cytokinesis [43], MLCK may also have a crucial role in this process. Indeed, treatment of both crane-fly and *Drosophila *spermatocytes with the MLCK inhibitor ML-7 reversibly blocked cytokinesis ([29]; this study). Similarly, ML-7 interferes with cytokinesis in sea urchin embryos [44] and with maintenance of F-actin in the contractile ring in mammalian cells [45]. Strikingly, ML-7 caused cleavage furrow regression and redistribution of actin filaments in a manner similar to U73122 and ET-18-OCH_3 _(this report). Furthermore, treatment with all three inhibitors caused loss of phosphorylated myosin regulatory light chain (phospho-Sqh) from the equator of the cell. Although ML-7 is reported to have other targets *in vitro *(*e.g*., PKA and PKC; [46]), the most straightforward interpretation of our results is that all three inhibitors interfere with cytokinesis by blocking MLCK activity, myosin regulatory light chain phosphorylation and myosin II contractility.

## Conclusion

We showed that two different PLC inhibitors, U73122 and ET-18-OCH_3_, have similar effects on cleavage furrow stability, F-actin localization and myosin regulatory light chain phosphorylation, indicating a role for PLC activity in cleavage furrow ingression. Moreover, inhibition of MLCK with ML-7 affects cytokinesis in a similar manner, suggesting that PIP_2 _hydrolysis and Ca^2+ ^release stimulate non-muscle myosin II to maintain actin-myosin interactions and stability of the contractile ring.

## Methods

### Live spermatocyte preparation

Preparation of crane-fly spermatocytes in fibrin clots has been described in detail elsewhere [47]; a modified protocol was used to examine *Drosophila *spermatocytes [10]. Briefly, testes from fourth instar crane-fly (*Nephrotoma suturalis *(Loew)) or third instar *Drosophila melanogaster *were dissected in Insect Ringer's buffer (0.13 M NaCl, 5 mM KCl, 1 mM CaCl_2_, and 5 mM KH_2_PO_4 _and 7 mM Na_2_HPO_4_·7H_2_O, pH 6.8) and transferred to a small drop of 3–10% fibrinogen in Insect Ringer's buffer on a glass coverslip. Testes were then pierced with an insect needle, allowing the cells to flow out. After waiting ~30 seconds for the cells to settle, 2–5 μl of bovine thrombin was added to form a clot. The clot was inverted onto a drop of Insect Ringer's buffer in a perfusion chamber and sealed with wax or silicon grease. Cells were perfused with Insect Ringer's buffer (with or without pharmacological agents) throughout the experiment.

### Cell treatments

All experiments were performed at room temperature (25°C). Stock solutions of 0.01 M U73122 (1-[6-[[17 β-3-methoxyestra-1,3,5(10)-trien-17-yl]amino]hexyl]-1H-pyrrole-2,5-dione; Calbiochem) or U73343 (1-[6-[[17 β-3-methoxyestra-1,3,5(10)-trien-17-yl]amino]hexyl]-2,5-pyrrolidine-dione; Calbiochem), 0.03 M ET-18-OCH_3 _(1-O-Octadecyl-2-O-methyl-rac-glycero-3-phosphorylcholine; Biomol Research Laboratories, Inc., Plymouth Meeting, PA), 0.05 M ML-7 (1-[5-Iodonaphthalene-1-sulfonyl]-1H-hexahydro-l,4-diazepine·HCl; Sigma-Aldrich) or 6 M NEM (Sigma-Aldrich) were prepared in dimethylsulfoxide (DMSO) (Sigma-Aldrich) and stored at -20°C. Prior to treatment, aliquots of each drug were thawed and diluted to the appropriate final concentration with Insect Ringer's buffer. To ensure that any observed effects were not due to the solvent, control cells were treated with the same final concentration of DMSO.

### Phase-contrast and fluorescence microscopy

Live *Drosophila *spermatocytes were imaged using an upright Zeiss Axioplan 2 microscope with a Zeiss Apochromat 100× oil immersion objective lens (NA 1.4). Phase-contrast and fluorescent images were recorded with an Axiocam CCD camera using AxioVision software for image acquisition. Live crane-fly spermatocytes were followed using a phase-contrast inverted Nikon microscope with a Nikon 100× oil-immersion objective (NA 1.3). Real-time images were recorded on videotape or on DVD.

### Immunostaining and fluorescence microscopy

Immunostaining and imaging of crane-fly and *Drosophila *spermatocytes were performed essentially as described [48] except that *Drosophila *spermatocytes were fixed with 4% paraformaldehyde (Electron Microscopy Sciences) rather than glutaraldehyde. *Drosophila *spermatocytes were stained with Alexa 488 phalloidin (Molecular Probes) for F-actin, 4',6-diamidino-2-phenylindole (DAPI; Molecular Probes) for DNA and 1:400 rabbit polyclonal antibody against phospho-Ser19-Sqh (from Luke Alphey [31]) and were mounted in Fluorescence Mounting Medium (Daxocytomation). Crane-fly spermatocytes were stained using 2.2 μM Alexa 488 phalloidin for F-actin (Molecular Probes) and 1:4000 YL1/2 rat monoclonal antibody against tyrosinated tubulin [46] and were mounted in Mowiol. Immunofluorescence images of *Drosophila *spermatocytes were collected with a Zeiss Axioplan 2 microscope and Axiovision 4.2 software. Confocal images of crane-fly spermatocytes were collected with Fluoview (Olympus) software, and were processed further using Image J (public domain software available at ) and Adobe Photoshop. All illustrations were prepared using Adobe Photoshop and were adjusted for image presentation only (brightness, contrast).

### Analysis of data

Cleavage furrow ingression was measured from individual frames extracted from time-lapsed recordings using custom software and plotted using SlideWrite as described previously [9,10].

## Abbreviations

DAG diacylglycerol

DMSO dimethylsulfoxide

ET-18-OCH_3 _1-O-Octadecyl-2-O-methyl-rac-glycero-3-phosphorylcholine; edelfosine (PLC inhibitor)

F-actin filamentous actin

IP_3 _inositol trisphosphate

ML-7 1-[5-Iodonaphthalene-1-sulfonyl]-1H-hexahydro-l,4-diazepine·HCl (MLCK inhibitor)

MLCK myosin light chain kinase

NEM N-ethylmaleimide

PI phosphatidylinositol

PIP phosphatidylinositol 4-phosphate

PIP_2 _phosphatidylinositol 4,5-bisphosphate

PLC phospholipase C

Sqh Spaghetti squash (*Drosophila *myosin regulatory light chain)

U73122 1-[6-[[17 β-3-methoxyestra-1,3,5(10)-trien-17-yl]amino]hexyl]-1H-pyrrole-2,5-dione (PLC inhibitor)

U73343 1-[6-[[17 β-3-methoxyestra-1,3,5(10)-trien-17-yl]amino]hexyl]-2,5-pyrrolidine-dione (inactive isomer of U73122)

## Authors' contributions

RW designed and carried out the *Drosophila *experiments and drafted the manuscript. LF designed and carried out the crane-fly experiments and commented on the manuscript. AF participated in the design of the study, analysis of the data, and helped edit the manuscript. JAB conceived the study, participated in its design and edited the manuscript. All authors read and approved the final manuscript.

## Supplementary Material

Additional file 1 Meiosis in a crane-fly spermatocyte. Time-lapse phase-contrast micrographs of a dividing crane-fly primary spermatocyte. Times are hr:min:s. Movie corresponds to Figure 2A. Click here for file

Additional file 2 Meiosis in a *Drosophila *spermatocyte. Time-lapse phase-contrast micrographs of a dividing *Drosophila *primary spermatocyte. Mitochondria and parafusorial membranes appear dark and outline the spindle during meiosis. Times are hr:min:s:ms. Movie corresponds to Figure 2B. Click here for file

Additional file 3 Inhibition of PLC with U73122 causes cleavage furrow regression in a dividing crane-fly spermatocyte. Dividing crane-fly spermatocyte treated with 4.3 μM U73122 at 1 min 36 sec (14:51:00). Note that this cell was followed for only a short time after treatment. Times are hr:min:s. Movie corresponds to Figure 2C. Click here for file

Additional file 4 Inhibition of PLC with U73122 causes cleavage furrow regression in a dividing *Drosophila *spermatocyte. Dividing *Drosophila *spermatocyte treated with 10 μM U73122 at 3 min 20 sec and washed at 18 min 20 sec. Times are hr:min:s:ms. Movie corresponds to Figure 2D. Click here for file

Additional file 5 Inhibition of PLC with ET-18-OCH_3 _causes cleavage furrow regression in a dividing crane-fly spermatocyte. Dividing crane-fly spermatocyte treated with 35 μM ET-18-OCH_3 _at 13:32:00. Times are hr:min:s. Movie corresponds to Figure 3A. Click here for file

Additional file 6 Inhibition of PLC with ET-18-OCH_3 _causes cleavage furrow regression in a dividing *Drosophila *spermatocyte. Dividing *Drosophila *spermatocyte treated with 35 μM ET-18-OCH_3 _at 5 min. 35 sec. Times are hr:min:s:ms. Movie corresponds to Figure 3B. Click here for file

Additional file 7 The alkylating agent NEM inhibits cytokinesis in a dividing crane-fly spermatocyte. Dividing crane-fly spermatocyte treated with 1 mM NEM at 15:02:00. Times are hr:min:s. Movie corresponds to Figure 4A. Click here for file

Additional file 8 The alkylating agent NEM inhibits cytokinesis in a dividing *Drosophila *spermatocyte. Dividing *Drosophila *spermatocyte treated with 1 μM NEM at 2 min 21 sec. Times are hr:min:s:ms. Cell treated similarly is shown in Figure 4B. Click here for file

Additional file 9 Inhibition of MLCK with ML-7 causes cleavage furrow regression in a dividing *Drosophila *spermatocyte. Dividing *Drosophila *spermatocyte treated with 80 μM ML-7 at 3 min 30 sec. After the cell is washed at 4 min 55 sec, furrowing resumes. Times are hr:min:s:ms. Cell treated similarly is shown in Figure 5A. Click here for file
